# High density lipoproteins and extracellular vesicles—distinct but overlapping circulating particles and their role in atherosclerosis

**DOI:** 10.3389/fcvm.2026.1831078

**Published:** 2026-07-29

**Authors:** Linas Černiauskas, Goda Aleknavičiūtė, Skaistė Arbačiauskaitė, Nicolas Mytzka, Susann Allelein, Inga Bikulčienė, Dovilė Karčiauskaitė

**Affiliations:** 1 Department of Physiology, Biochemistry, Microbiology and Laboratory Medicine, Institute of Biomedical Sciences, Vilnius University, Vilnius, Lithuania; 2 Biomarker Research Laboratory, Translational Health Research Institute, Faculty of Medicine, Vilnius University, Vilnius, Lithuania; 3 Neuroscience Research Center, Charité-Universitätsmedizin Berlin, Berlin, Germany; 4 MicroDiagnostics Unit, Fraunhofer Institute for Cell Therapy and Immunology IZI, Leipzig, Germany; 5 Institute of Clinical Immunology, Medical Faculty, Leipzig University, Leipzig, Germany; 6 Department of Preclinical Research, Centre for Innovative Medicine, Vilnius, Lithuania

**Keywords:** atherosclerosis, cardiovascular disease, circulating nanoparticles, extracellular vesicle cargo, extracellular vesicles, extracellular vesicle isolation, high-density lipoproteins, lipoprotein-extracellular vesicle interactions

## Abstract

Cardiovascular disease remains the leading cause of morbidity and mortality worldwide, with atherosclerosis representing its principal pathological basis. High-density lipoproteins (HDL) and extracellular vesicles (EVs) are abundant circulating particles implicated in lipid metabolism, vascular inflammation, intercellular communication, and thrombotic processes relevant to atherosclerosis. Although HDLs and EVs differ in origin, structure, biogenesis, and canonical function, they share overlapping physicochemical and molecular features. Their density ranges substantially overlap, and small EV populations may approach the upper size range of HDL particles, making their separation from plasma technically challenging. As a result, common isolation workflows may generate HDL-enriched or EV-enriched fractions rather than fully particle-specific preparations, complicating the interpretation of proteomic, lipidomic, nucleic acid, and functional studies. This review compares the structural and biophysical characteristics, biogenesis pathways, molecular cargo, and atherosclerosis-related functions of HDLs and EVs. We highlight how both particle classes contribute to endothelial activation, inflammation, cholesterol handling, foam cell formation, plaque progression, and thrombosis, while also emphasizing their distinct biological roles. Finally, we discuss whether HDL–EV overlap should be interpreted solely as methodological co-isolation or may also reflect biologically relevant interactions within circulating nanoparticle networks in atherosclerosis.

## 1 Introduction

Cardiovascular disease (CVD) remains the leading cause of morbidity and mortality globally, with 19.2 million deaths reported in 2023 (1). Atherosclerosis, the principal pathological process underlying many cardiovascular events, is a chronic inflammatory disease driven by lipid accumulation in the arterial wall (2). Because lipids, inflammation, and intercellular communication are central to atherogenesis, circulating nanoparticles have become an important focus in efforts to understand disease mechanisms and identify clinically useful biomarkers.

Historically, lipoproteins and extracellular vesicles (EVs) emerged from separate fields of research. Lipoproteins became central to cardiovascular biology after low-density lipoproteins (LDL) and their lipid cargo were established as causal factors in atherosclerotic cardiovascular disease (3). High-density lipoproteins (HDL) were subsequently studied as potentially atheroprotective particles, although the failure of HDL-cholesterol-raising strategies to consistently reduce cardiovascular events shifted attention from HDL concentration to HDL composition and function (4–12). EVs, in contrast, were initially viewed largely as cellular debris but are now recognized as biologically active particles involved in intercellular communication and disease-associated signaling (13–16).

Although HDLs and EVs are biologically distinct, their study in plasma is complicated by overlapping physicochemical properties. HDL particles are dense lipoproteins with a phospholipid monolayer, apolipoprotein scaffold, and hydrophobic lipid core, whereas EVs are cell-derived particles enclosed by a phospholipid bilayer membrane (15–19). Despite these structural differences, their density ranges substantially overlap, and the smallest EV populations approach the upper end of the HDL particle size range (15–17, 20–22). In plasma, where lipoproteins and soluble proteins greatly outnumber EVs, this overlap creates a persistent challenge for isolating, characterizing, and interpreting downstream molecular or functional assays (23, 24). This problem is not only methodological. HDL-associated apolipoproteins, including ApoA-I, ApoC-III, and ApoE, have been detected in EV preparations, while lipoprotein carryover is frequently reported in EV isolation workflows (25–29). These observations may reflect technical co-isolation, but they may also indicate biologically relevant interactions, including protein corona formation on EV surfaces (25, 29). Therefore, strict separation of HDLs and EVs is essential when assigning cargo or function to a specific particle class, but co-isolated fractions may also contain information about physiological nanoparticle interactions that should not be dismissed as contamination without context (16, 24–27).

This review examines HDLs and EVs as distinct but overlapping circulating particles in atherosclerosis. Rather than treating overlap solely as an analytical artifact, we consider how shared physical properties, partially overlapping molecular cargo, and possible particle–particle interactions complicate the interpretation of HDL- and EV-associated effects. Thus, a central question emerging from this review is whether HDL–EV overlap should be treated primarily as an isolation artefact, or whether co-isolated HDL- and EV-associated molecules may also reflect biologically meaningful interactions between circulating particles in atherosclerosis.

## 2 Circulating nanoparticle landscape

Human blood contains many highly heterogeneous nanoparticles, including lipoproteins [chylomicrons and their remnants, very low-density lipoproteins, intermediate-density lipoproteins, low-density lipoproteins, lipoprotein(a), high-density lipoproteins], extracellular vesicles, non-vesicular extracellular particles (NVEPs), including exomeres and supermeres, and soluble protein complexes. These particles participate in processes ranging from lipid transport and intercellular communication to inflammation, vascular homeostasis, and dysfunction. Since their initial isolation by Macheboeuf in 1929 and subsequent separation from other serum lipoproteins by Gofman and colleagues using ultracentrifugation, HDL particles have been studied primarily in lipid transport and cardiovascular disease (30, 31). In parallel, EV research originated from studies of blood coagulation, beginning with the “particulate fraction” described by Chargaff and West in the 1940s and later termed “platelet dust” by Peter Wolf in the 1960s (14). Although initially regarded as cellular debris, EVs are now recognized as actively released particles produced by virtually all cell types and involved in intercellular communication across diverse biological systems (14, 32–34). Despite the historically different fields of research for HDL particles and extracellular vesicles, accumulating evidence suggests that these particles may coexist within a heterogeneous plasma nanoparticle environment. As a result, more attention has been directed toward the overlapping physicochemical properties of HDL particles and EVs, which complicate their isolation, characterization, and interpretation in biomedical studies.

**Figure 1:**
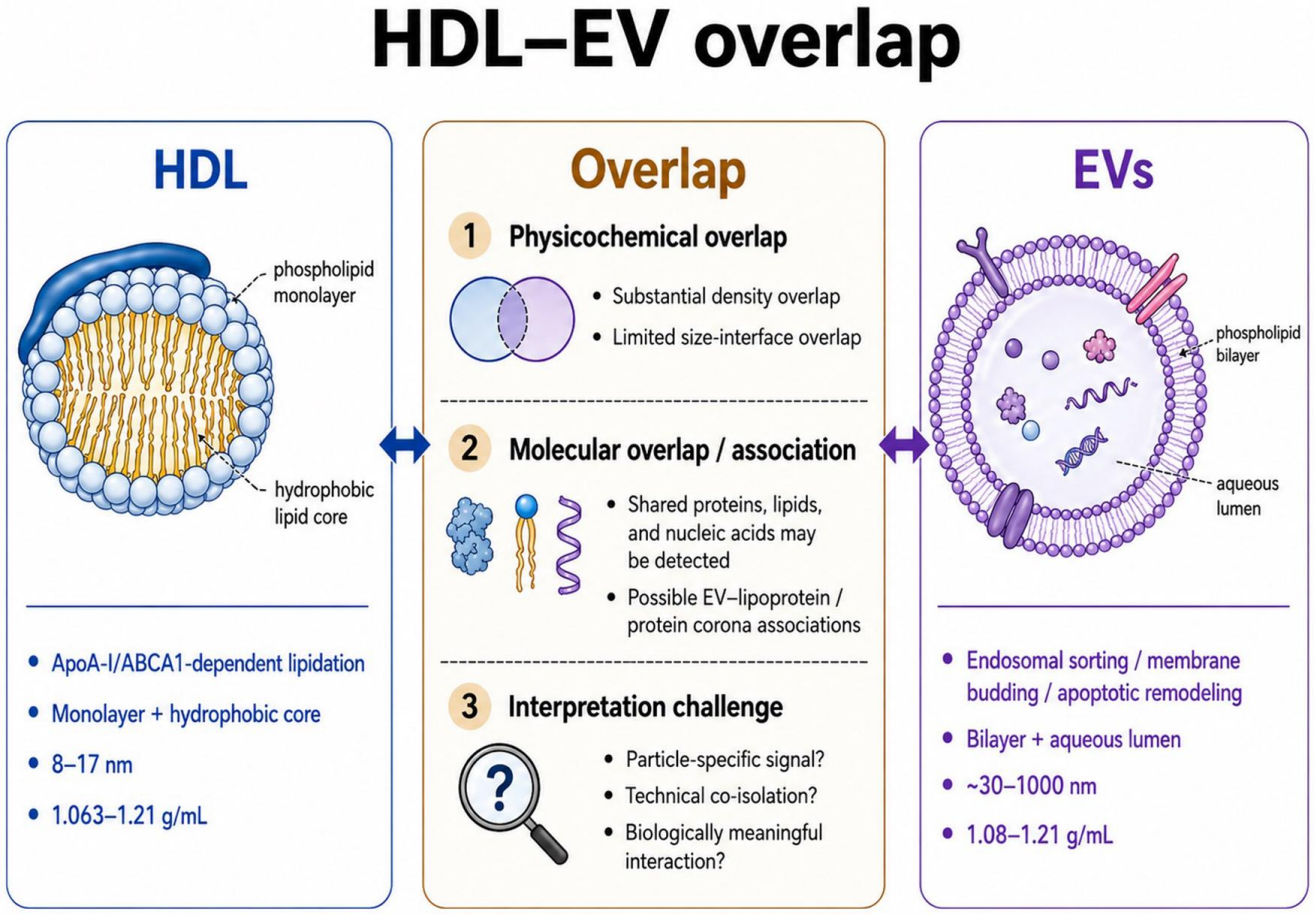
HDL-EV overlap as a challenge for analytical and biological interpretation.

Both HDL particles and EVs are highly heterogeneous particle populations comprising multiple subtypes with partially overlapping physicochemical properties. HDL particles are small spherical or discoidal lipoproteins with a density of 1.063–1.21 g/mL and a typical diameter of 8–17 nm. Based on size, density, and morphology, HDL particles can be further classified into small, nascent, poorly lipidated discoidal particles and larger, lipid-rich spherical particles. By contrast, ultracentrifugation-based classification most commonly separates them into HDL2 (density 1.063–1.125 g/mL) and HDL3 (density 1.125–1.21 g/mL) (17). Whereas EVs are generally larger particles, ranging from approximately 30 to 1,000 nm, and may display spherical or non-spherical morphologies; however, their reported density range of 1.08–1.21 g/mL overlaps substantially with that of HDL particles (15, 16, 20, 21, 35). Thus, HDL-EV overlap is most pronounced in density and at the interface between larger HDL particles and the smallest EV populations, rather than across the full EV size spectrum. In accordance with Minimal Information for Studies of Extracellular Vesicles 2023 (MISEV2023), EVs are defined here as cell-released, lipid bilayer-delimited particles that cannot replicate. Comparable heterogeneity is observed among EV populations, although EV classification remains more difficult because particle size, density, morphology, and marker expression do not reliably define biogenesis (16, 19). Recently described extracellular nanoparticle fractions, non-vesicular extracellular particles (NVEPs), including exomeres (30–50 nm) and supermeres (15–25 nm), further expand the spectrum of circulating extracellular particles and reinforce the analytical complexity of plasma and serum particle preparations. Therefore, throughout this review, we preferentially use operational descriptors based on measurable features, including particle size, density, surface markers, cellular source, and isolation method. These overlapping physicochemical and molecular features are summarized in Figure 1.

These physicochemical overlaps explain why HDL particles and EVs are frequently co-isolated from plasma or serum. Size-based methods, including size-exclusion chromatography and ultrafiltration, enrich particles according to hydrodynamic radius but cannot fully resolve HDL particles, particularly larger HDL subclasses, from the smallest EVs and NVEPs. Density-based methods, including ultracentrifugation and density gradient centrifugation, face an even greater limitation because the density range of HDL substantially overlaps with that of EVs. This problem is amplified by the high abundance of circulating lipoproteins and soluble plasma proteins relative to EVs, which increases the likelihood of HDL carryover into EV preparations and EV-associated signals within HDL-enriched fractions (16, 22, 24, 26, 29, 36). Importantly, HDL-EV overlap should not be interpreted exclusively as a methodological artifact. In biological fluids, EVs acquire a protein and lipoprotein corona that can influence their biodistribution, uptake, and biological activity (25). Therefore, co-isolated HDL particles, apolipoproteins, and EVs may reflect both incomplete analytical separation and physiologically relevant particle interactions (25, 26, 37, 38). Collectively, this heterogeneity and partial overlap in size and density complicate the analytical separation of circulating nanoparticles and may confound the interpretation of functional, omics-based, and biomarker studies in cardiovascular disease.

## 3 Biogenesis and molecular architecture

The growing recognition of HDL-EV overlap has increased interest in whether their shared physicochemical characteristics reflect convergent biological functions or primarily result from methodological overlap during particle isolation and analysis. Although HDL particles and EVs may partially overlap in biological fluids, they are generated through fundamentally distinct pathways (39, 40). HDL particles arise through ApoA-I/ATP-binding cassette transporter A1 (ABCA1)-dependent lipidation and subsequent enzymatic remodeling in circulation, whereas EVs originate from endosomal sorting, or plasma membrane budding, or apoptotic membrane remodeling. These biogenetic differences determine their molecular organization, cargo-loading mechanisms, structural stability, and biological functions.

The formation of nascent discoidal HDL particles initiates primarily at the surfaces of hepatocytes and enterocytes and requires ApoA-I, ABCA1, and phospholipids. Binding of lipid-poor ApoA-I to ABCA1 promotes phospholipid and cholesterol efflux, membrane curvature, and assembly of nascent discoidal HDL particles (41–45). After release into circulation, nascent HDL undergoes further remodeling: phospholipid transfer protein (PLTP) contributes to phospholipid transfer, lecithin-cholesterol acyltransferase (LCAT) esterifies free cholesterol to cholesteryl esters that accumulate in the hydrophobic core and promote spherical HDL maturation, and cholesteryl ester transfer protein (CETP) subsequently remodels HDL by exchanging cholesteryl esters and triglycerides with ApoB-containing lipoproteins (46–49). Thus, HDL biogenesis and maturation are driven by lipidation, lipid transfer, and enzymatic remodeling.

EV biogenesis is more heterogeneous and depends on the EV subtype and the parental cell's physiological state. Many small EVs arise from inward budding of the endosomal membrane, generating intraluminal vesicles within multivesicular bodies, which are released as EVs upon fusion with the plasma membrane (50, 51). The endosomal sorting complexes required for transport (ESCRT) machinery, including ESCRT-0, I, II, and III complexes and adaptor proteins such as ALG-2-interacting protein X (ALIX) and syntenin, contributes to cargo sorting and intraluminal vesicle formation (51, 52). ESCRT-independent mechanisms also contribute to EV formation, including ceramide-rich membrane domains and tetraspanin-enriched microdomains that involve CD63, CD81, and CD9 (51–54). In contrast, microvesicles arise through outward budding of the plasma membrane, involving Ca^2^⁺-dependent phospholipid scrambling, phosphatidylserine externalization, cytoskeletal remodeling, and actomyosin contraction, whereas apoptotic bodies are released during apoptosis as a consequence of membrane blebbing and increased hydrostatic pressure (19, 53). Therefore, EV biogenesis reflects dynamic membrane remodeling and cargo sorting processes that are strongly influenced by cell type, activation state, stress, and injury.

### 3.1 Molecular architecture and structural organization

HDL particles have two main structural compartments: a phospholipid monolayer surface and a hydrophobic lipid core (18). The surface monolayer contains phospholipids and free cholesterol, with hydrophilic head groups oriented toward the aqueous environment and hydrophobic acyl chains oriented toward the lipid core. Apolipoproteins and enzymes embedded or associated with this surface layer contribute to HDL stability, lipid exchange, and biological function (17, 18, 27). ApoA-I is the principal structural apolipoprotein and is central to HDL assembly, lipid solubilization, and particle remodeling (42, 43, 55, 56). The HDL core contains mainly cholesteryl esters and triglycerides, together with smaller amounts of hydrophobic bioactive lipids and lipid-soluble molecules (18).

In contrast, EVs are enclosed by a phospholipid bilayer membrane surrounding an aqueous lumen. This bilayer architecture allows EVs to transport both membrane-associated cargo and protected luminal cargo, including proteins, lipids, metabolites, and nucleic acids (16, 23, 57, 58). The lipid bilayer protects luminal cargo from enzymatic degradation, whereas transmembrane proteins, tetraspanins, integrins, adhesion molecules, and other surface-associated proteins contribute to EV stability, targeting, uptake, and signaling (16, 23, 59). In biological fluids, EVs can also acquire a protein and lipoprotein corona that modulates phagocytic clearance, biodistribution, immune recognition, and biological activity (25, 37). EV membrane properties are further influenced by lipid composition, protein-to-lipid ratio, curvature, stiffness, and the cellular origin of the parental cell (54, 59).

These architectural differences are central to distinguishing HDL particles from EVs. HDL particles are organized as phospholipid monolayer lipoprotein structures optimized for lipid solubilization, lipid exchange, and the transport of hydrophobic molecules (17). EVs are membrane-bound vesicles optimized for the transfer of membrane-associated and luminal molecular cargo between cells (16). Consequently, shared size or density ranges do not imply shared biogenesis or identical biological function. Rather, the overlap between HDL particles and EVs creates an analytical challenge, while their distinct architectures support different, though potentially interacting, roles (26). Therefore, HDL-associated data may primarily reflect lipoprotein remodeling and lipid transport capacity, whereas EV-associated data may reflect the activation state and injury responses of vascular and immune cells in atherosclerosis. At the same time, HDL-EV co-isolation and possible particle–corona interactions mean that functional readouts in plasma may reflect both distinct particle biology and their interactions within the circulating nanoparticle environment (25, 29).

## 4 Shared principles of molecular cargo transport

HDL particles and EVs both function as circulating carriers of bioactive molecular cargo, including proteins, lipids, metabolites, and nucleic acids. However, their cargo-loading principles differ substantially. HDL cargo composition is shaped primarily by ApoA-I-dependent particle formation, lipid exchange, enzymatic remodeling, and acquisition of proteins and lipids from plasma and other lipoproteins (39). In contrast, EV cargo reflects the biogenesis pathway, membrane composition, parental-cell identity, activation state, and corona formation (25, 51, 53). This distinction is particularly important in cardiovascular disease, where inflammatory remodeling can simultaneously alter HDL composition and EV release, thereby complicating the attribution of specific cargoes to either particle class (29, 60, 61).

### 4.1 Protein cargo and proteomic overlap

Proteomic studies have revealed substantial complexity and heterogeneity in both HDL and EV cargo composition, particularly in pathways related to lipid metabolism, inflammation, immunity, and cardiovascular disease (62–66).

HDL particles contain a dynamic proteome dominated by ApoA-I and ApoA-II, which comprise approximately 85%–90% of the HDL protein mass and serve as the HDL structural scaffold (67). ApoA-I is the dominant HDL protein, accounting for up to 70% of HDL protein mass, and each HDL particle is estimated to contain between two and five ApoA-I molecules, depending on particle size (55, 56). Owing to its amphipathic helices, ApoA-I is essential for the solubilization and incorporation of phospholipids into HDL particles (55). ApoA-II represents the second most abundant HDL-associated protein, comprising approximately 15%–20% of HDL protein mass, although it is present only in a subset of HDL particles (56). Additional apolipoproteins associated with HDL include ApoA-IV, C-I, C-II, C-III, C-IV, D, E, F, L, H, and M, which contribute directly to HDL remodeling and function (17, 68–70). For example, ApoC-I inhibits CETP activity and thereby influences HDL lipid composition by increasing cholesteryl ester (CE) accumulation within the HDL lipid core (69) (69), while ApoA-IV and ApoM are proposed carriers of sphingosine-1-phosphate (S1P), a bioactive lipid involved in endothelial barrier protection (70, 71). HDL particles additionally transport enzymes involved in lipid metabolism, including CETP, lecithin-cholesterol acyltransferase (LCAT), and phospholipid transfer protein (PLTP), which participate in lipid transfer and HDL maturation (47–49). Proteomic studies have also identified complement proteins, immunoglobulins, acute phase proteins, coagulation factors, and protease inhibitors associated with HDL particles (63, 72, 73). Notably, HDL particles become enriched with serum amyloid A (SAA) during the acute phase response together with a concomitant reduction in ApoA-I content (60, 74). Such compositional remodeling is increasingly recognized as a hallmark of dysfunctional HDL in systemic inflammation and cardiovascular disease (20, 74).

Like HDL particles, EVs contain a highly diverse proteome that contributes to vesicle stability, trafficking, signaling, and interactions with recipient cells (65, 75). The Vesiclepedia database currently contains proteomic data from more than 3,500 studies comprising over 500,000 protein entries, highlighting the remarkable molecular complexity of EVs. Among the most consistently identified EV-associated proteins are tetraspanins, annexins, cytoskeletal proteins, signaling proteins, metabolic enzymes, and heat shock proteins (66). Tetraspanins such as CD9, CD63, CD81, CD82, and CD151 contribute to the formation of tetraspanin-enriched microdomains involved in membrane organization, adhesion, membrane fusion, and signaling (50, 66, 75). Annexins participate in EV formation, membrane stability, and interactions with target cells (76–78), whereas cytoskeletal proteins reflect the involvement of cytoskeletal remodeling during EV biogenesis and secretion (53). EVs additionally transport signaling proteins, metabolic enzymes, and heat shock proteins that participate in signal transduction, cellular metabolism, stress responses, and protein stabilization (66, 79, 80).

Increasing evidence suggests apparent proteomic overlap between HDL particles and EVs. EV-enriched fractions have been reported to contain several apolipoproteins, including ApoA-I, ApoB, ApoC-III, and ApoE (25, 28, 29, 65, 66). The detection of ApoA-I in EV-associated fractions raises the possibility of lipid-related functions, including cholesterol handling, but whether EV-associated ApoA-I retains HDL-like activity remains unresolved (25, 26, 29). Similarly, EV-associated ApoC-III and ApoE may reflect pathways related to triglyceride metabolism and cholesterol clearance, although their functional activity in EV-associated fractions requires further validation (25, 28, 81, 82). Collectively, these findings suggest that HDL particles and EVs may participate in partially overlapping lipid transport and inflammatory pathways. However, whether such overlap reflects methodological co-isolation, EV-associated corona formation, biologically meaningful lipoprotein–EV interactions, or mixed nanoparticle assemblies remains unclear.

### 4.2 Lipid cargo and lipidomic overlap

Three pioneering studies combining chromatographic and mass spectrometry techniques provided reference measurements of the HDL lipidome in human participants (83–85). Most of the HDL lipidome consists of four main lipid groups: neutral lipids, phospholipids, sphingolipids, and minor lipids (18). Although EV research has developed extensively over the past two decades, plasma EV lipidomics remains less standardized than HDL lipidomics. Most studies have been conducted on EVs isolated from human cell lines or from individual groups under various health conditions, resulting in substantial variability in reported lipid profiles (54, 59, 86–89). Unlike HDL particles, EVs have a lipid bilayer membrane, and their lipid composition varies with EV size, cellular origin, disease status, isolation workflow, and analytical platform (87).

HDL particles and EVs share several lipid classes, including cholesterol, phospholipids, sphingolipids, glycosphingolipids, and minor lipid species (18, 54, 83, 84, 86–88). However, the key difference is not the absolute presence or absence of these lipids but their relative abundance, structural localization, and functional context. In HDL particles, lipids are arranged in a surface monolayer and a hydrophobic core, whereas in EVs, lipids form a bilayer membrane that contributes to vesicle stability, cargo protection, membrane interaction, and uptake by recipient cells (17 86–89).

Neutral lipids, particularly free cholesterol (FC) and cholesterol esters (CE), are central to HDL lipid transport. FC is located in the HDL surface layer, whereas CE accumulates in the hydrophobic core after LCAT-mediated esterification (18, 83, 84). In EVs, FC contributes to endosomal membrane organization and EV biogenesis by influencing membrane fluidity, curvature, and vesicle-generating domains (51, 54). However, the detection of FC, CE, triacylglycerols (TG), or diacylglycerols (DAG) in EV-enriched plasma fractions may also indicate co-isolation with lipoproteins and incomplete separation of EVs from lipid-rich plasma particles (24, 26, 29, 87).

Phospholipids form the HDL surface monolayer and are important for cholesterol movement between cells and HDL, including HDL-mediated cholesterol efflux and cholesterol flux mediated by scavenger receptor class B type I (SR-BI) (90). Phosphatidylcholine (PC) is the most abundant HDL phospholipid, whereas other phospholipids may contribute to specific HDL properties, including surface charge and interactions with lipases or membrane proteins (18, 63, 83, 84, 91). Phospholipids are major components of the EV lipidome. In EVs, Phosphatidylserine (PS) is particularly important because its externalization provides a negatively charged membrane surface that promotes EV clearance, immune recognition, coagulation enzyme complex assembly, and thrombin generation (23, 54, 87, 92).

Sphingolipids are another important, shared lipid group. In HDL particles, sphingomyelin (SM) contributes to surface-layer rigidity, while sphingosine-1-phosphate (S1P) is mainly transported in plasma by ApoM-containing HDL and has been linked to endothelial barrier regulation and vascular protection (71, 85, 93, 94). HDL also contains ceramides (Cer) and glycosphingolipids, although Cer is transported less efficiently than SM and S1P (83, 91, 93, 95). In EVs, Cer and glycosphingolipids contribute to membrane organization, vesicle formation, membrane microdomains, and EV uptake (96–98). Beyond EV biology, ceramide- and glycosphingolipid-related pathways have been implicated in vascular inflammation, nitric oxide signaling, cellular stress responses, and atherosclerotic cardiovascular disease (99, 100).

Minor lipids are also relevant to HDL and EV lipidomic studies. Free fatty acids (FFA) and F2-isoprostanes (IsoP) have been identified in HDL (91, 101), while EVs may contain FFA, oxysterols, acylcarnitines, and other low-abundance lipid metabolites that may reflect cellular metabolic or inflammatory states (54, 102).

In atherosclerosis, the HDL- and EV-associated lipid species most relevant to particle overlap are those involved in lipid transport, membrane organization, inflammation, endothelial regulation, coagulation, and biomarker interpretation. Neutral lipids are central to this overlap: FC and CE participate in HDL-mediated cholesterol transport, whereas detection of FC, CE, TG, or DAG in EV-enriched plasma fractions may reflect true EV lipid cargo, co-isolated HDL or other lipoproteins, or both (24, 29, 83, 84, 87). Phospholipids, including PC, lysophosphatidylcholine (LPC), and plasmalogen species, contribute to HDL surface organization, cholesterol efflux, and HDL functionality, while HDL phospholipid profiles have been shown to distinguish acute coronary syndrome from stable coronary artery disease (90, 91, 103). In EVs, externalized PS provides a negatively charged membrane surface that supports EV clearance, immune recognition, coagulation enzyme complex assembly, and thrombin generation, thereby contributing to EV-associated procoagulant activity (23, 54, 87, 92). Sphingolipids further connect HDL and EV functions: SM contributes to membrane rigidity, Cer and hexosylceramides (HexCer) are involved in membrane curvature, EV biogenesis, and sphingolipid signaling, lactosylceramides (LacCer) and gangliosides participate in membrane microdomain organization and EV uptake, and HDL-associated S1P is linked to endothelial barrier regulation, vascular protection, and limitation of vascular inflammation (85, 91, 93, 94, 96–100). Finally, low-abundance lipid species, including IsoP, oxysterols, acylcarnitines, FFA, and other oxidized lipid species, may contribute to oxidative stress, inflammation, metabolic signaling, and biomarker interpretation in cardiovascular disease (54, 101–103).

Overall, the shared lipid species do not necessarily imply a shared function. In plasma-derived fractions, overlapping lipidomic signatures may indicate true biological remodeling, lipid exchange between circulating particles, EV–lipoprotein association, or residual lipoprotein co-isolation. This limitation is especially relevant for biomarker and functional studies in atherosclerosis because HDL lipid composition itself is disease-related, and lipid species identified in EV-enriched samples may represent EV cargo, HDL/lipoprotein cargo, or both. Accurate EV characterization and assessment of lipoprotein co-isolation are therefore required before assigning a lipidomic signal to EV-specific biological activity (22, 24, 29).

### 4.3 Other bioactive molecules

Despite their traditional role in lipid transport, HDL particles have been proposed as carriers of lipid-soluble vitamins, hormones, small RNAs (sRNAs), and various metabolites. Alpha-tocopherol (Vitamin E), known for its antioxidant properties, has been shown to accumulate in HDL particles and be transported by these particles to human retinal epithelial cells (104). Vitamin A precursors, such as carotenoids, including alpha-carotene, beta-carotene, and cryptoxanthin, have also been linked to HDL particles (105). In several studies, HDL has been shown to bind hormones due to interactions between specific apolipoprotein sites and hormones. These hormones include thyroxine, estradiol, pregnenolone, and dehydroepiandrosterone (106, 107). Various studies have demonstrated that HDL particles can transport genetic material, including sRNAs such as microRNAs (miRNAs) (108–111). A particularly relevant example is miR-223, which has been reported in HDL-associated RNA cargo and implicated in endothelial regulation, including modulation of inflammatory adhesion pathways (108). EV-associated miRNAs, including miR-126 and miR-155, have also been linked to endothelial repair or activation, leukocyte recruitment, and atherosclerosis-related inflammation (112, 113). These examples illustrate that HDL particles and EVs may carry overlapping classes of regulatory RNAs, but the modes of transport, cellular origins, delivery efficiencies, and functional consequences may differ substantially. Thus, HDL-associated miRNAs may contribute to gene regulation, although their exact role in RNA transport and recipient-cell regulation remains incompletely defined.

Given that HDLs can mediate the transfer of various regulatory molecules, EVs provide a structurally distinct but functionally related system for transporting biologically active compounds between cells. In addition to their ability to transfer various proteins and lipids, there is substantial evidence that EVs are involved in nucleic acid transport. EVs have been shown to encapsulate messenger RNA (mRNA), miRNA, long non-coding RNA (lncRNA), ribosomal RNA (rRNA), transfer RNA (tRNA), and, in cases of larger EVs, mitochondrial DNA (mtDNA), and single- and double-stranded genomic DNA molecules (112–114). These molecules are not merely cargo but can also exert regulatory effects on recipient cells, influencing their gene expression and signaling pathways and ultimately affecting their phenotype, inflammatory and immune responses, and even proliferation under physiological and pathological conditions (115–117). Beyond nucleic acids, pioneering studies have identified bioactive metabolites and intermediates in EV cargo. For instance, bioactive lipid intermediates and eicosanoids have been identified in myeloid and tumor cell-derived EVs (40). EVs not only contain eicosanoids and their precursors but also the enzymes necessary for their production, enabling them to generate these bioactive molecules once released into the extracellular matrix. The various eicosanoids produced by EVs can modulate inflammation, regulate vascular cell function and immune cell recruitment, and influence EV uptake (40).

Taken together, HDL particles and EVs are structurally distinct circulating nanoparticles but show substantial overlap in molecular cargo, including apolipoproteins, enzymes, complement-related proteins, lipids, small RNAs, and metabolites. This overlap may reflect technical co-isolation, association with the EV corona, lipoprotein–EV interactions, or shared involvement in vascular inflammatory and metabolic pathways. The main overlapping and particle-enriched cargo classes are summarized in Table 1.

**Table 1:** Molecular overlap between HDL particles and EVs.

Cargo	HDL-associated components	EV-associated components	Shared/overlapping elements	Interpretation caveat
Proteins	ApoA-I, ApoA-II, ApoC-III, ApoE, ApoM, enzymes, complement, SAA	Tetraspanins, annexins, enzymes, ApoA-I, ApoB, ApoC-III, ApoE, corona proteins	ApoA-I, ApoC-III, ApoE, SAA, complement-related proteins	True cargo *vs.* corona *vs*. co-isolation
Lipids	CE, FC, TG, DAG, PC, LPC, SM, S1P, Cer	FC, CE, TG, DAG, PC, LPC, PS, SM, Cer, HexCer, LacCer	FC, CE, TG, DAG, PC, LPC, SM, Cer	Biological overlap *vs*. lipoprotein carryover
Nucleic acids	sRNAs, miRNAs	miRNA, mRNA, lncRNA, rRNA, tRNA, mtDNA	miRNA	Carrier attribution requires separation controls
Other bioactive molecules	Vitamins, hormones, metabolites	Metabolites, eicosanoids	Metabolites	Limited comparability across methods

ABCA1, ATP-binding cassette transporter A1; ApoA-I, apolipoprotein A-I; ApoA-II, apolipoprotein A-II; ApoB, apolipoprotein B; ApoC-III, apolipoprotein C-III; ApoE, apolipoprotein E; ApoM, apolipoprotein M; CE, cholesteryl esters; Cer, ceramides; DAG, diacylglycerols; EVs, extracellular vesicles; FC, free cholesterol; FFA, free fatty acids; HDL, high-density lipoprotein; HexCer, hexosylceramides; LacCer, lactosylceramides; LCAT, lecithin-cholesterol acyltransferase; lncRNA, long non-coding RNA; LPC, lysophosphatidylcholine; miRNA, microRNA; mRNA, messenger RNA; mtDNA, mitochondrial DNA; PC, phosphatidylcholine; PLTP, phospholipid transfer protein; PS, phosphatidylserine; S1P, sphingosine-1-phosphate; SAA, serum amyloid A; SM, sphingomyelin; sRNA, small RNA; TG, triacylglycerols.

## 5 High-density lipoprotein and extracellular vesicle functional interplay in atherosclerosis

Atherosclerosis is a chronic lipid-driven inflammatory disease in which endothelial dysfunction, leukocyte recruitment, lipid accumulation, foam cell formation, plaque progression, and thrombosis develop through interconnected vascular and immune mechanisms (2, 118, 119). HDL particles and EVs influence many of these processes through distinct but context-dependent mechanisms. HDL became central to atherosclerosis research after the inverse association between HDL cholesterol (HDL-C) and cardiovascular risk led to the “HDL hypothesis,” which viewed HDL as intrinsically atheroprotective. However, HDL-C-raising strategies, including niacin and CETP inhibitors, failed to consistently reduce cardiovascular events, and Mendelian randomization studies did not support a causal protective role for HDL-C concentration alone (6, 7, 120–122). This shifted the field toward HDL functionality, particle composition, and disease-associated remodeling. Functional HDL supports cholesterol efflux, endothelial integrity, antioxidant activity, anti-inflammatory signaling, and antithrombotic regulation, whereas inflammatory remodeling can generate dysfunctional HDL with reduced protective capacity and potentially pro-atherogenic properties (8, 74, 123, 124). EVs are not uniformly pro- or anti-atherogenic; rather, they function as context-dependent mediators of intercellular communication. By transferring proteins, lipids, metabolites, and nucleic acids between vascular, immune, and circulating cells, EVs can amplify endothelial activation, leukocyte recruitment, macrophage responses, and plaque progression under inflammatory conditions, while EVs released in homeostatic or reparative settings may support vascular repair and tissue communication (13, 51, 112, 125, 126).

Importantly, HDL particles and EVs may not act as entirely independent circulating entities but may participate in dynamic biological interactions within the circulation. Both transport bioactive lipids, proteins, and regulatory RNAs, and emerging evidence suggests that direct EV-lipoprotein interactions may influence particle biodistribution, cellular uptake, and biological activity (127). Therefore, the overlap between these particles has important methodological implications for interpreting atherosclerosis studies. Shared proteins, lipids, and nucleic acids detected in HDL- or EV-enriched fractions may represent particle-specific cargo, EV corona components, biologically meaningful EV-HDL interactions, or co-isolation during size- or density-based workflows (24–26, 29, 110, 128). Accordingly, functional readouts should be interpreted in relation to particle purity, isolation strategy, and evidence level. In this chapter, we compare HDL and EV contributions across key stages of atherosclerosis, from endothelial dysfunction and leukocyte recruitment to foam cell formation, plaque progression, thrombosis, and acute coronary syndromes, distinguishing mechanistic, animal-model, and human evidence where possible.

### 5.1 Endothelial dysfunction and early vascular inflammation

Endothelial dysfunction represents one of the earliest detectable events in atherogenesis and is characterized by impaired endothelial barrier integrity, reduced nitric oxide (NO) bioavailability, increased oxidative stress, and enhanced expression of adhesion molecules and proinflammatory mediators (2, 118, 129).

Under physiological conditions, HDL particles exert several vasoprotective effects on endothelial cells. HDL-associated ApoA-I interacts with scavenger receptor class B type I (SR-BI), activating phosphoinositide 3-kinase (PI3 K)/Akt -dependent endothelial nitric oxide synthase (eNOS) signaling and promoting NO production and vasodilation (130). In parallel, ApoM-bound sphingosine-1-phosphate (S1P) carried by HDL activates endothelial S1P receptors, particularly sphingosine-1-phosphate receptor 1 (S1PR1) and, in some contexts, sphingosine-1-phosphate receptor 3 (S1PR3), supporting endothelial junction integrity, reducing vascular permeability, and maintaining barrier function (71, 94). HDL particles can also suppress endothelial inflammatory activation, including reduced expression of vascular cell adhesion molecule-1 (VCAM-1), intercellular adhesion molecule-1 (ICAM-1), and monocyte chemoattractant protein-1 (MCP-1), thereby limiting leukocyte adhesion and transendothelial migration (108, 124, 131). However, under certain metabolic and inflammatory conditions, HDL particles undergo compositional and functional remodeling, including enrichment with SAA, oxidized lipids, and ceramides, as well as depletion or functional impairment of protective components such as ApoA-I and S1P (60, 73, 74, 132, 133). Metabolic and inflammatory remodeling converts HDL into dysfunctional particles that lose their endothelial-protective properties and, under certain pathological conditions, can promote endothelial dysfunction and pro-inflammatory signaling (74).

EVs also regulate endothelial activation and vascular inflammation during early atherogenesis. Monocyte-derived EVs can activate Toll-like receptor 4 (TLR4)/nuclear factor-*κ*B (NF-*κ*B) signaling in endothelial cells, increasing ICAM-1 and C–C motif chemokine ligand 2 (CCL2)/MCP-1 expression, which promotes leukocyte adhesion, monocyte chemotaxis, and transendothelial recruitment, thereby amplifying vascular inflammation (134, 135). In addition, nicotine- or smoking-associated EV-miRNA signaling has also been linked to endothelial dysfunction, whereas neutrophil-derived EVs can promote monocyte transendothelial migration through miR-155-dependent induction of ICAM-1, VCAM-1, and CCL2 (136). Conversely, in mice, endothelial EVs enriched in miR-126 have been shown to support vascular repair by promoting endothelial regeneration through sprouty-related EVH1 domain-containing protein 1 (SPRED1)-dependent pathways. However, this protective effect is impaired under hyperglycemic or metabolic stress conditions (137).

Together, these findings suggest that inflammatory remodeling of both HDL particles and EVs contributes to endothelial dysfunction during early atherogenesis. Their overlapping effects on endothelial signaling further complicate attribution of particle-specific functions in cardiovascular disease.

### 5.2 Leukocyte recruitment and immune activation

Leukocyte recruitment is a crucial step by which endothelial activation develops into sustained inflammation within the arterial wall. Adhesion molecules such as VCAM-1, ICAM-1, E-selectin, and *P*-selectin guide leukocyte rolling, firm adhesion, and transmigration into the subendothelial space (2, 118). Recruited monocytes then differentiate into macrophages and help maintain the inflammatory environment that drives plaque progression. HDL particles and EVs can influence these events by modulating endothelial–leukocyte interactions, cytokine signaling, and immune cell communication.

HDL-associated ApoA-I attenuates monocyte activation and inflammatory cytokine production while promoting cholesterol efflux through ABCA1- and ATP-binding cassette transporter G1 (ABCG1) -dependent pathways (8–10, 138, 139). Maintenance of macrophage cholesterol homeostasis is important because cholesterol accumulation alters membrane lipid raft organization and amplifies inflammatory receptor signaling, including Toll-like receptor (TLR)-dependent pathway. Through these mechanisms, HDL-mediated cholesterol efflux can reduce NF-*κ*B activation and production of proinflammatory cytokines such as TNF-α, IL-1β, and IL-6 (119). *In vitro* and *in vivo* studies have shown that HDL and ApoA-I can suppress chemokine and chemokine receptor expression, thereby limiting monocyte chemotaxis and recruitment (131). In addition, HDL-associated bioactive lipids, particularly ApoM-bound S1P, may contribute to immune cell trafficking and vascular immune homeostasis (93, 94). HDL can also suppress cholesterol crystal-induced inflammasome activation in experimental macrophage systems, although the extent to which this mechanism operates in human atherosclerotic plaques remains less certain (140). Similarly, in murine models, HDL has been reported to promote less inflammatory macrophage phenotypes, but this effect appears context- and model-dependent (141). However, inflammatory remodeling substantially alters the immunomodulatory properties of HDL particles. Dysfunctional HDL particles enriched with serum amyloid A, oxidized phospholipids, and ceramides may enhance inflammatory signaling within vascular and immune cells and impair HDL-mediated suppression of leukocyte recruitment (74, 85, 93, 95, 142).

EVs also contribute to leukocyte recruitment and immune activation during atherogenesis. EVs released from activated endothelial cells, platelets, monocytes, macrophages, and neutrophils can carry adhesion molecules, cytokines, chemokines, bioactive lipids, and inflammatory miRNAs that modulate immune cell behavior and amplify vascular inflammation (143–145). For instance, plaque-derived EVs can transfer ICAM-1 to endothelial cells, thereby promoting monocyte adhesion and transendothelial migration (115). Platelet-derived EVs may promote leukocyte adhesion and recruitment through P-selectin-and chemokine-mediated pathways (115, 146). Human monocyte-derived EVs can stimulate endothelial activation and induce the expression of adhesion molecules and cytokines via TLR4/NF-*κ*B signaling, thereby increasing endothelial expression of ICAM-1, VCAM-1, CCL2/MCP-1, and related inflammatory mediators (147). Neutrophil-derived EVs can facilitate monocyte recruitment by delivering miR-155 to atheroprone endothelium, inducing endothelial expression of ICAM-1, VCAM-1, and CCL2 (112).

Inflammatory remodeling shifts the balance from HDL-mediated immune regulation toward EV-driven propagation of vascular inflammation. Consequently, coordinated changes in both particle populations may promote persistent leukocyte recruitment and plaque progression.

### 5.3 Lipid accumulation and foam cell formation

Lipid accumulation in the arterial *intima* and subsequent foam cell formation are hallmark events in the early development of atherosclerotic plaques. Retention and modification of apolipoprotein B-containing lipoproteins within the subendothelial space promote monocyte recruitment, macrophage differentiation, and uncontrolled lipid uptake through scavenger receptor-mediated pathways (2, 118, 148). Both HDL particles and EVs can regulate macrophage cholesterol homeostasis, inflammatory signaling, and foam-cell formation.

HDL particles are central facilitators of reverse cholesterol transport (RCT) as ApoA-I-mediated cholesterol efflux through ABCA1- and ABCG1-dependent pathways represents a key antiatherogenic mechanism that limits cholesterol accumulation within macrophages (8, 10, 138). Mature HDL particles subsequently transport cholesterol to the liver for excretion either directly through scavenger receptor class B type I (SR-BI)-mediated uptake or indirectly through CETP-dependent transfer to ApoB-containing lipoproteins (7, 138). Cholesterol efflux capacity (CEC), which measures the initial step of RCT, is inversely associated with atherosclerotic burden and has been linked to incident cardiovascular events, supporting its value as a functional measure of HDL-mediated atheroprotection (8–10, 138). HDL-mediated cholesterol efflux alters plasma membrane lipid raft composition, thereby modulating pathways associated with TLR activation, inflammasome assembly, NF-*κ*B signaling, and cytokine production (119). These mechanisms link cholesterol handling directly to macrophage inflammatory phenotype and innate immune activation. HDL particles may also reduce LDL oxidation and limit the formation of oxidized LDL, a major driver of macrophage lipid uptake, foam cell formation, and vascular inflammation (11, 12, 148–151). However, HDL functionality becomes substantially impaired during chronic inflammatory and metabolic disorders. Dysfunctional HDL particles exhibit reduced CEC. This change can diminish HDL-mediated protection against lipid accumulation and may promote inflammatory signaling within macrophages (8, 119). Therefore, HDL-C concentration alone does not adequately reflect HDL function, and HDL quality appears more relevant than quantity for assessing its effects on foam-cell formation and plaque progression.

Whereas HDL generally limits foam-cell formation by promoting cholesterol efflux, EVs can either promote or reduce macrophage lipid accumulation depending on their origin and cargo. EVs released from macrophages, platelets, endothelial cells, adipose tissue, and hepatocytes can carry inflammatory mediators, oxidized lipids, apolipoproteins, and regulatory miRNAs that modulate macrophage cholesterol metabolism and inflammatory activation (143–145). Platelet-derived EVs have been shown to increase macrophage uptake of oxidized LDL and inflammatory cytokine production, potentially enhancing foam-cell formation (152). Adipose tissue-derived EVs also influence macrophage cholesterol handling: visceral adipose tissue exosomes from obese mice promote foam-cell formation and M1-like polarization by reducing ABCA1/ABCG1-mediated cholesterol efflux, whereas perivascular adipose tissue-derived exosomes may reduce foam-cell formation by decreasing scavenger receptor-mediated oxidized LDL uptake and increasing ABCA1/ABCG1-dependent cholesterol efflux (153, 154). Several EV-associated miRNAs have been implicated in the regulation of cholesterol homeostasis and foam-cell formation, e.g., steatotic hepatocyte-derived small EVs enriched in miR-30a-3p, which suppress ABCA1-mediated cholesterol efflux (155). In contrast, other EV populations may exert protective effects by delivering anti-inflammatory or cholesterol-regulating cargoes. For example, M2 macrophage-derived exosomes enriched in miR-7683-3p enhance cholesterol efflux through the peroxisome proliferator-activated receptor *γ* (PPAR*γ*)/liver X receptor *α* (LXR*α*)/ABCG1 pathway, highlighting the context-dependent nature of EV-mediated regulation during atherosclerosis (156).

Together, these findings highlight that HDL particles and EVs converge in regulating macrophage cholesterol homeostasis despite their distinct biological origins.

### 5.4 Plaque progression and chronic inflammation

As atherosclerotic lesions progress, persistent lipid accumulation and unresolved inflammation drive the transition from early fatty streaks to complex fibroatheromatous plaques characterized by necrotic core formation, smooth muscle cell remodeling, extracellular matrix reorganization, and progressive immune cell infiltration (2, 118).

Beyond regulating cholesterol homeostasis, HDL particles modulate chronic plaque inflammation by suppressing macrophage activation, reducing oxidative stress, and promoting inflammation-resolving pathways. HDL and ApoA-I inhibit activation of the NLR family pyrin domain-containing 3 (NLRP3) inflammasome and reduce the production of pro-inflammatory cytokines, thereby limiting persistent vascular inflammation and necrotic core expansion (140). HDL also influences macrophage polarization toward a less inflammatory phenotype and attenuates matrix metalloproteinase expression, processes associated with improved plaque stability and reduced extracellular matrix degradation (157). However, these protective activities are progressively lost during inflammatory remodeling, contributing to plaque progression and instability (74).

EVs actively contribute to plaque progression by propagating inflammatory signals between endothelial cells, macrophages, vascular smooth muscle cells (VSMCs), and other cells within the plaque microenvironment. Macrophage-derived EVs stimulate pro-inflammatory signaling and matrix remodeling, promoting vascular inflammation and plaque destabilization by transferring cytokines, proteases, and regulatory miRNAs (158). VSMC-derived EVs further contribute to plaque progression by promoting vascular calcification and extracellular matrix remodeling, processes associated with increased plaque complexity and instability (159). Together, these findings indicate that EVs not only reflect chronic vascular inflammation but also actively participate in the remodeling processes that drive plaque progression.

The balance between functional HDL and disease-associated EV populations is an important determinant of plaque stability. Coordinated inflammatory remodeling of both particle classes may therefore contribute to persistent inflammation, extracellular matrix degradation, and progressive plaque destabilization.

### 5.5 Plaque rupture, thrombosis, and acute coronary syndromes

As atherosclerotic plaques progress toward advanced and unstable phenotypes, chronic inflammation, expansion of the necrotic core, extracellular matrix degradation, and endothelial erosion collectively increase the likelihood of plaque rupture and thrombus formation (2, 118).

Under physiological conditions, HDL particles exhibit antithrombotic and vasculoprotective effects. HDL can inhibit platelet activation and aggregation by modulating platelet membrane cholesterol content, suppressing intracellular calcium signaling, and enhancing endothelial nitric oxide (NO) bioavailability (123, 130). HDL-associated ApoA-I and S1P additionally support endothelial integrity and reduce endothelial activation, thereby limiting exposure of procoagulant subendothelial components to circulating blood (70, 93). HDL has also been reported to influence secondary hemostasis by enhancing the anticoagulant activities of activated protein C and protein S, which promote the inactivation of coagulation factors Va and VIIIa (160). Experimental and human observational studies have further associated favorable HDL characteristics with reduced thrombin generation, lower endogenous thrombin potential, and enhanced fibrinolytic activity (161, 162).

In contrast to the predominantly protective functions attributed to physiological HDL particles, several inflammation-associated EV populations can promote thrombosis and plaque destabilization during advanced atherosclerosis. Platelet-derived EVs represent prominent circulating EV populations in cardiovascular disease and can provide procoagulant membrane surfaces enriched in phosphatidylserine (145). Externalized phosphatidylserine supports assembly of coagulation enzyme complexes and thrombin generation, whereas functional tissue factor expression on EVs appears more context- and disease-dependent (92). EVs released from inflammatory endothelial cells, activated platelets, macrophages, and apoptotic vascular cells may therefore contribute to local and systemic procoagulant activity during unstable atherosclerotic disease (143, 145). Beyond coagulation, EVs may contribute to plaque destabilization by transferring inflammatory and remodeling-associated cargo between vascular and immune cells. Macrophage-, platelet-, endothelial-, and vascular smooth muscle cell-derived EVs can influence smooth muscle cell phenotype, extracellular matrix remodeling, calcification, and inflammatory signaling within vulnerable plaques (115, 145, 163). By transferring regulatory miRNAs, oxidized lipids, phosphatidylserine-rich membranes, and coagulation-related mediators, EVs may amplify structural and inflammatory changes that favor plaque instability. Human clinical-association studies suggest that circulating EV profiles change during acute coronary syndromes (ACS). EV concentration, surface epitope profiles, and EV-associated lipid signatures have been reported to differ in patients with acute myocardial infarction (145). In a pilot study of ST-elevation myocardial infarction (STEMI), circulating EVs were enriched in sphingolipids, including ceramides, dihydroceramides, and sphingomyelins, and these signatures correlated with high-sensitivity troponin concentrations and decreased after reperfusion therapy (164). EV-associated cardiac miRNAs, including miR-1, miR-208, and miR-499, have also been investigated in myocardial injury settings, although their clinical utility remains exploratory (165).

Progression to plaque rupture is driven not only by structural weakening of the plaque but also by profound changes in intercellular signaling and coagulation, processes that are strongly influenced by both dysfunctional HDL and procoagulant EVs.

## 6 Isolation challenges and methodological considerations

The substantial overlap between high-density lipoproteins (HDL) and extracellular vesicles (EVs) represents one of the major methodological challenges in cardiovascular nanoparticle research. The overlap among these nanoparticles complicates the isolation of HDL- or EV-enriched fractions from plasma and serum and may confound proteomic, lipidomic, transcriptomic, biomarker, and functional studies in atherosclerosis.

### 6.1 Clinical and Pre-analytical variables

For robust cross—study comparisons of circulating nanoparticles as biomarkers, transparent reporting of clinical and pre-analytical conditions is crucial. In general, clinical studies should clearly define the cohort, diagnostic criteria, recruitment setting, fasting state, medication use, and major comorbidities, as these factors can strongly influence circulating nanoparticles. Studies should account for age, sex, smoking status, diabetes, lipid-lowering therapy, and other cardiovascular risk factors, as these represent major confounders in atherosclerosis research (166). Pre-analytical variables influence both EV and HDL measurements, although their relative importance differs between the two particle populations, highlighting the need for standardized sample collection, preparation, and processing. The number of platelet-derived EVs changes due to platelet activation, artificially increasing EV counts and altering vesicle composition. Consequently, the International Society on Thrombosis and Haemostasis (ISTH) recommends a double-centrifugation protocol of two subsequent centrifugation steps at 2,500 × g for 15 min to obtain platelet-free plasma from blood samples that should be processed within 2 h after sample collection (167). In contrast, anticoagulant choice appears to have a comparatively smaller effect on EV concentration and size distributions under standardized conditions (168). Storage and limited freeze–thaw exposure have also been reported to exert relatively minor effects on EV concentration and particle size when samples are processed and stored appropriately at −80 °C (169). For HDL analysis, the most influential pre-analytical variables are sample type, anticoagulant selection, and overall processing quality. Serum and plasma are not always interchangeable because clotting, platelet release, and tube additives can alter lipid and protein composition, thereby affecting HDL-related measurements (170–172). Proper centrifugation and sample clarification are also essential, as residual cells, platelets, and triglyceride-rich lipoproteins may interfere with HDL quantification and characterization. Storage conditions and repeated freeze–thaw cycles can compromise lipoprotein integrity and assay performance, particularly in studies assessing HDL composition and function rather than cholesterol concentration alone (8, 170, 173). Although platelet activation does not directly alter HDL particles, it can indirectly influence HDL measurements by altering the plasma matrix and releasing bioactive components during clotting.

Given the substantial overlap in particle size and physicochemical properties between EVs and lipoproteins, standardization of sample collection, prompt processing, controlled centrifugation, minimization of platelet activation, and avoidance of repeated freeze–thaw cycles are essential for reliable assessment of both particle classes.

### 6.2 Isolation strategies for HDLs and EVs

A wide range of isolation methods is currently used for HDL and EV separation, each with distinct strengths and limitations. Differential ultracentrifugation (UC) is widely used and provides relatively high particle recovery, but it frequently co-isolates EVs, lipoproteins, protein aggregates, and other plasma nanoparticles. The UC protocol for EVs, which is not standardized and shows variable g-forces and centrifugation times, is generally considered the most critical factor, as it strongly affects vesicle recovery and size distribution (16, 174). Additionally, standard differential UC leaves substantial numbers of EVs and HDL in the supernatant and biases recovery toward larger particles, while extended spins at >100,000 g promote aggregation, deformation, and possible fusion, thereby influencing the quantitative yield, apparent size distribution, and molecular composition of the resuspended EVs and lipoproteins (175).

Density gradient ultracentrifugation (DGC) improves separation compared to differential UC, but the overlapping density ranges of EVs and HDL particles limit complete separation (16, 29). Furthermore, DGC protocols differ substantially, including top-down vs. bottom-up loading, the use of a high-density cushion, differences in gradient medium, volume, and concentration, centrifugation times and speeds, and sample and gradient volumes. These parameters affect particle yield, purity, and the relative recovery efficiency of EVs and HDL vs. other lipoproteins and soluble proteins (176). Although the MISEV guidelines emphasize detailed reporting of all methodological parameters to enhance transparency and reproducibility, the resulting diversity of non-standardized DGC workflows makes it difficult to directly compare EV- or HDL-enriched fractions across published studies (16). Additionally, most DGC workflows are labor-intensive and have low throughput.

Ultrafiltration (UF) is commonly used for concentration of cell culture-derived EVs as it is operationally simple and scalable. However, ultrafiltration alone does not efficiently separate EVs from soluble proteins, protein aggregates, or lipoproteins. Therefore, for complex specimens such as plasma, which are used in cardiovascular research, UF should not be used as a standalone isolation method but rather combined with additional pre- or post-purification steps (177).

Size-exclusion chromatography (SEC) is increasingly used for plasma EV studies because it preserves particle integrity, reduces soluble protein contamination, and is relatively reproducible owing to commercial columns, standardized protocols, and a simple workflow (16). However, SEC alone does not yield pure EVs or HDL; larger lipoproteins [very-low-density lipoproteins (VLDL), chylomicron remnants, and a subset of LDL] co-elute with EVs in early fractions, while most soluble proteins, protein aggregates, and small lipoproteins, including HDL, elute in later fractions (36, 174). Limited loading capacity and partial adsorption of vesicles and proteins to the matrix also lead to moderate recovery and substantial dilution (174).

Immunoaffinity and depletion-based approaches can improve enrichment or removal of selected particle populations. Capture of EV-associated markers such as CD9, CD63, CD81, or cell-type-specific epitopes may enrich defined EV subpopulations, whereas depletion of ApoA-I-, ApoB-, or ApoE-positive material may reduce lipoprotein contamination (16, 174). However, these approaches are intrinsically marker-dependent and may exclude biologically relevant subpopulations that lack the selected target (174). Additionally, the elution of captured EVs frequently requires harsh or poorly standardized conditions that may alter EV integrity or surface composition. Conversely, ApoA-I depletion may remove both free HDL particles and biologically meaningful EV–lipoprotein complexes or EV corona components (25). Therefore, immunoaffinity strategies are most appropriate when the target population is well-defined, and loss of marker-negative particles is acceptable.

Higher-resolution, multimodal workflows are increasingly important for cardiovascular nanoparticle research to overcome the limitations of the single-isolation methods mentioned above (16, 174). SEC followed by density gradient centrifugation, iodixanol density gradients followed by SEC or bind-elute chromatography, and density-based lipoprotein depletion before EV isolation can improve EV purity and reduce lipoprotein-associated background (26). Asymmetric flow field-flow fractionation (AF4) provides high-resolution separation of extracellular nanoparticles across broad size ranges and has been used to distinguish EV subsets from exomeres and other non-vesicular particles (178). Microfluidic, ion-mobility, resistive-pulse sensing, high-sensitivity flow cytometry, and single-particle imaging or proteomic approaches may further improve separation and characterization, to uncover previously undetected HDL-EV interactions and provide deeper insight into HDL-EV crosstalk (127). However, multistep isolation workflows further complicate cross-study comparability, as no universally accepted “gold standard” protocol or standardized combinations currently exist. Moreover, each additional isolation step can introduce selection biases and cumulative particle losses, thereby altering the apparent distribution of cargo across nanoparticle classes. Consequently, nominally similar “EV” or “HDL” fractions can represent substantially different particle compositions in different studies. Existing method papers predominantly compare distinct isolation principles, whereas large, parametric studies that vary conditions within a single method (such as g-forces, rotor types, gradient architectures, or buffer composition) are less common. Nevertheless, published data indicate that even modest protocol changes can alter yield, selectively enrich, or deplete specific subpopulations, and shift omics profiles (179–181).

The choice of methodology should be guided by the intended downstream application, with assay validation incorporating technical replicates and internal controls to ensure data reliability and reproducibility. For isolated EV proteomics and lipidomics, strict reduction of HDL and other lipoproteins is critical; combined SEC–density gradient, density-based lipoprotein depletion followed by SEC, or AF4-based workflows are preferable to single-step ultracentrifugation or precipitation. For HDL proteomics and lipidomics, workflows should include assessment of EV-associated markers and, where needed, EV-depletion or orthogonal fractionation to exclude vesicular contamination.

### 6.3 Best practices and future directions

Current guidelines emphasize transparent reporting and orthogonal characterization rather than reliance on a single isolation method. MISEV2023 recommends detailed reporting of pre-analytical variables, separation procedures, recovery and specificity, quantification approaches, marker validation, and method limitations (16). For plasma and serum studies, characterization should include EV-associated markers, lipoprotein markers, abundant plasma protein contaminants, particle morphology, size distribution, and single-particle or orthogonal validation when possible. Recommended analytical controls include EV-positive markers such as tetraspanins and cytosolic EV-associated proteins, lipoprotein markers such as ApoA-I, ApoB, ApoE, and ApoC-III, and non-vesicular protein contaminants such as albumin and immunoglobulins. Particle counting by nanoparticle tracking analysis (NTA) or tunable resistive pulse sensing (TRPS) should not be interpreted as EV-specific without biochemical or single-particle validation, because these methods cannot distinguish EVs from lipoproteins or other nanoparticles by size alone (16, 174).

A practical framework is therefore needed. Strict HDL-EV separation is essential when the aim is to assign a molecular cargo or biological function specifically to HDL particles or EVs (16, 26). This applies particularly to proteomics, lipidomics, RNA studies, mechanistic uptake assays, cholesterol efflux assays, and functional assays of coagulation or inflammation (29, 36). In contrast, preservation of partially co-isolated native nanoparticle assemblies may be informative when the aim is to study plasma nanoparticle ecology, EV corona biology, lipoprotein-EV interactions, or biomarker signatures that reflect the circulating state rather than a purified particle class (25, 38). Thus, HDL particles and EVs should be considered biologically distinct entities that require rigorous separation for particle-specific attribution, yet their co-isolation may also reflect physiologically meaningful interactions under inflammatory and atherosclerotic conditions (Table 2).

**Table 2:** Application-specific separation and interpretation framework.

Research aim	Recommended strategy	Required controls/validation	What can be concluded	Main limitation
EV cargo attribution	SEC + DGC/density cushion or AF4-based fractionation. Consider ApoA-I/ApoB/ApoE depletion only if corona loss is acceptable	EV markers (CD9/CD63/CD81 and ALIX/TSG101) lipoprotein/protein controls: ApoA-I, ApoB, ApoE, ApoC-III, albumin/IgG; recovery, particle size/morphology, EV-poor counter-fractions	EV-enriched or EV-associated cargo	Do not claim “EV-specific” unless supported by orthogonal validation. Stringent purification may remove physiological corona or HDL-EV complexes
HDL cargo attribution	HDL enrichment by UC, immunoaffinity, or SEC-based lipoprotein fractionation, with parallel EV-marker assessment	HDL/lipoprotein markers: ApoA-I, ApoA-II, Contamination controls: ApoB/ApoE, EV markers (CD9/CD63/CD81 and ALIX/TSG101), EV-rich counter-fraction	HDL-enriched or HDL-associated cargo	EV/corona carryover may mimic HDL-associated cargo. ApoA-I positivity alone does not prove HDL specificity
Functional assays (CEC, inflammatory index, endothelial uptake, coagulation, immune activation)	Analyze matched EV-rich, HDL-rich, and protein/lipoprotein-rich fractions; include depletion/add-back where feasible	Counter-fractions, assay-specific normalization by particle number, protein, ApoA-I, cholesterol, or EV-marker signal; EV/lipoprotein markers	Fraction-associated activity, particle causality only with depletion/add-back or orthogonal fractionation	Normalization strategy can change interpretation, functional activity may arise from mixed nanoparticle assemblies
Biomarker studies (reproducibility and clinical associations are the priority)	Fixed SOP, parallel fraction profiling where feasible, maximal purity not always required if clearly reported	Sample type, anticoagulant, processing time, platelet depletion, hemolysis/platelet activation, storage, freeze–thaw cycles, recovery, specificity	Fraction-associated, compartment-associated, or mixed nanoparticle-associated biomarkers	Reproducible association does not prove particle specificity, pre-analytical variation may dominate signal
HDL-EV interaction biology (native complexes are the object of study)	Preserve complexes using gentle SEC/co-fractionation or mild density workflows, avoid aggressive depletion as primary workflow	Co-fractionation across orthogonal methods, reciprocal capture/depletion of ApoA-I/ApoB/ApoE and EV markers, imaging or single-particle analysis, functional validation	Candidate HDL-EV association, biological relevance only if functionally validated	Must distinguish true interaction, corona association, co-isolation, and post-isolation artefact

EV, extracellular vesicle; HDL, high-density lipoprotein; SEC, size-exclusion chromatography; DGC, density gradient centrifugation; AF4, asymmetric flow field-flow fractionation; UC, ultracentrifugation; SOP, standard operating procedure; CEC, cholesterol efflux capacity; Apo, apolipoprotein; ALIX, ALG-2-interacting protein X; TSG101, tumor susceptibility gene 101 protein; CD, cluster of differentiation; IgG, immunoglobulin G.

Future studies should move toward standardized, application-specific workflows. At minimum, studies should report sample type, anticoagulant, time to processing, centrifugation steps, platelet depletion, assessment of hemolysis or platelet activation, storage conditions, freeze–thaw cycles, isolation workflow, recovery, specificity, and marker panels (16, 174). For cardiovascular studies, reporting should additionally include lipoprotein contamination metrics, such as ApoA-I, ApoB, ApoE, cholesterol, triglycerides, and albumin, together with EV markers and functional validation (16). Additionally, studies would benefit from parallel analysis of multiple fractions derived from the same sample (e.g., EV-enriched, HDL-enriched, and protein-rich fractions), as the resulting counter-fractions provide internal controls that increase the interpretive power of a given dataset and allow omics and functional readouts to be more confidently attributed to specific nanoparticle classes. Such a roadmap would improve reproducibility, enable comparisons across studies, and clarify whether observed HDL- or EV-associated effects in atherosclerosis reflect particle-specific biology, technical co-isolation, or biologically meaningful HDL-EV interactions.

## 7 Conclusions and future directions

HDLs and EVs are biologically distinct circulating particles with different origins, structures, and biogenesis pathways. However, their overlapping density and size ranges, and shared molecular associations make independent study difficult, particularly in plasma, where lipoproteins and soluble proteins greatly exceed EVs in abundance.

In atherosclerosis, this distinction is important because both particle classes are linked to processes that contribute to disease initiation and progression. HDLs are mainly associated with cholesterol efflux, lipid transport, endothelial support, antioxidative and anti-inflammatory functions, and modulation of platelet and coagulation responses. EVs are more closely linked to intercellular communication, endothelial activation, leukocyte recruitment, macrophage lipid accumulation, foam cell formation, plaque progression, and acute coronary syndromes. These functions are not identical, but they converge on shared atherogenic pathways, especially vascular inflammation, lipid metabolism, and thrombotic complications.

This overlap complicates the interpretation of HDL- and EV-enriched preparations. When the aim is to assign specific cargo or function to one particle class, complementary separation and characterization approaches are essential, including parallel assessment of EV markers, apolipoproteins, lipids, and abundant plasma proteins. Without this, proteomic, lipidomic, nucleic acid, and functional readouts relevant to atherosclerosis may be incorrectly attributed to either HDLs or EVs. However, HDL-EV overlap should not automatically be treated as contamination. The presence of apolipoproteins and other lipoprotein-associated molecules in EV preparations may reflect technical co-isolation, but it may also indicate biologically relevant interactions, including protein corona formation. Such interactions could influence nanoparticle biodistribution, cellular uptake, cholesterol handling, inflammatory signaling, and thrombosis in atherosclerosis.

Future studies should therefore use application-specific workflows. Strict separation is required for particle-specific mechanistic claims, whereas co-isolated fractions may remain informative when studying circulating nanoparticle networks. Parallel analysis of HDL-enriched, EV-enriched, and protein-rich fractions, combined with functional validation, will be needed to determine whether the observed effects in atherosclerosis reflect HDL-specific, EV-specific, or physiologically meaningful HDL-EV interactions, or methodological carryover.
